# Gut microbiota and butyrate level changes associated with the long-term administration of proton pump inhibitors to old rats

**DOI:** 10.1038/s41598-019-43112-x

**Published:** 2019-04-29

**Authors:** Sun Min Lee, Nayoung Kim, Ryoung Hee Nam, Ji Hyun Park, Soo In Choi, Young-Tae Park, Yeon-Ran Kim, Yeong-Jae Seok, Cheol Min Shin, Dong Ho Lee

**Affiliations:** 1 Department of Internal Medicine, Seoul National University Bundang Hospital, Seongnam, Gyeonggi-do South Korea; 2 Department of Internal Medicine and Liver Research Institute, Seoul National University College of Medicine, Seoul, South Korea; 3 Department of Biological Sciences and Institute of Microbiology, Seoul National University, Seoul, South Korea; 4 Korea Institute of Science and Technology Natural Products Research Institute, Gangneung, South Korea

**Keywords:** Clinical microbiology, Clinical microbiology, Medical research, Microbiome

## Abstract

The association between adverse effects of PPI and gut microbiota in old age has yet to be elucidated. We assessed changes in the gut microbiota and butyrate levels following the long-term administration of PPIs to old rats and investigated their associations. F344 aged male rats were fed a PPI-supplemented diet for 50 weeks. The ileal microbiota was analysed by metagenomic sequencing of the 16S rRNA, while the butyrate concentration was measured by high-performance liquid chromatography. We observed a significant decrease in microbial diversity following PPI administration in the 2-year-old rats but not in the 74-week-old rats. PPI treatment reduced both commensal bacteria and opportunistic pathogens, particularly in the 2-year-old rats. Enterotypes comprising the majority of the control samples were enriched in *Lactobacillus*, while other enterotypes in the PPI group were dominated by *Turicibacter* or *Romboutsia*. The PPI treatment reduced the butyrate concentrations in the intestines and colons of 74-week-old rats compared to the control group. The abundance of *Lactobacillus* significantly correlated with butyrate concentrations in 74-week-old rats. In conclusion, long-term administration of PPIs alters the gut microbiota and butyrate concentrations in rats, particularly in old age, which may be an underlying mechanism of PPI-induced adverse effects such as pseudomembranous colitis.

## Introduction

Proton pump inhibitors (PPIs) induce gastric acid suppression and are commonly prescribed medications in the treatment of gastrointestinal disorders. The long term use of PPIs sometimes has structural or functional consequences, such as parietal cell hyperplasia, fundic gland polyps, enterochromaffin cell-like (ECL) cell hyperplasia, hyperchromograninaemia, hypochlorhydria and hypergastrinaemia^1^.

PPIs alter the composition of the gut microbiota and increase the risk of enteric bacterial infection, such as by *Clostridium difficile*^2–4^. Studies of human faecal samples have reported that the long-term use of PPIs (5–10 years) can moderately shift the gut microbiota composition^3,5^. The results of a crossover trial studying the effects of the short-term use of PPIs (four weeks) revealed alterations in specific taxa related to *C. difficile* infection and bacterial overgrowth, with observations of increased abundances of Enterococcaceae, Streptococcaceae, Micrococcaceae, and Staphylococcaceae and a decreased abundance of Clostridiales^6^. However, there have been contrasting results regarding whether PPIs significantly reduce gut microbial diversity. A significant reduction in gut microbial diversity was observed after long-term PPI administration^5^, while no significant changes were observed in other studies involving short- or long-term PPI administrations^3,6^. Due to these varying results, the ability of PPIs to potentially induce alterations in gut microbiota must be demonstrated with additional evidence. In addition, human studies using faecal samples have limitations in that it is difficult to control environmental factors such as diet, antibiotics, and sanitation, and that faecal samples contain mixed microbial components from whole gastrointestinal contents.

In older individuals, the gut microbiota appears to be less resistant to external stimuli such as dietary alterations^7,8^. Inflammaging and immunosenescence during ageing alter the gut environment and influence microbial growth, contributing to age-associated alterations in the gut microbiota^9^. In our previous study, we observed that the gut microbiota of old rats was more susceptible to high-fat diet-induced inflammation and colonic cell proliferations through assessments of microbial diversity and the Firmicutes/Bacteroidetes ratio^8^. However, a lack of evidence regarding the association between ageing and PPI-induced microbial changes prevented us from elucidating the risk of long-term PPI use in the elderly population. As a preliminary study, we investigated the effect of long-term PPI administration on gut microbiota of 74-week-old Fischer 344 (F344) rats (approximately 60 years in humans), and confirmed that the long-term administration of PPIs induced alterations in the microbiota of the terminal ileum of F344 rats^10^. In addition to these findings, the results of the present study further demonstrate the association between ageing and PPI-induced microbial changes by studying rats at a frailer age (104-week-old, equal to 2-year-old rats, approximately 80 years in humans) and by estimating changes in the concentrations of microbial products.

Major microbial changes associated with PPI usage include alterations in both luminal and mucosal bacterial taxa: Lachnospiraceae (unique to the lumen), Comamonadaceae, and *Clostridium* spp. in the oesophagus; *Helicobacter pylori* (unique to the mucosa) and Methylobacteriaceae in the stomach; Bifidobacteria in the small intestine; and *Lactobacillus* (unique to the lumen) and Bacteroidetes in the large intestine^11,12^. The major components of the luminal and mucosal microbiota have distinct functions. Luminal bacteria tend to affect the host through the production of metabolites, such as short-chain fatty acids (SCFAs) and gases, whereas adherent bacteria are more associated with mucosal immunity. In this study, we analysed the luminal microbiome and investigated the changes in SCFA production to gain insight into their possible impact on colonic health.

The microbial fermentation of dietary carbohydrate residues such as fibre in the large intestine results in the production of SCFAs^13^. Typical bacteria with saccharolytic metabolism with beneficial effects are Lactobacilli and Bifidobacteria^13^. Since most SCFAs are absorbed in exchange for bicarbonate, the microbial production of SCFAs and the neutralization by bicarbonate affect the luminal pH^11^. The decrease in pH from the ileum to the caecum due to changes in the SCFA concentration alters the gut microbiota composition and inhibits the overgrowth of pathogenic bacteria, such as Enterobacteriaceae and Clostridia^11^. Among SCFAs, butyrate has been studied extensively and may have a greater anticarcinogenic role than other SCFAs in the colon^14^. The present study investigated the level of butyrate in four intestinal regions, the duodenum, jejunum, ascending and descending colon, making this the first study to measure the PPI-induced changes in butyrate concentration in diverse regions of the small and large intestines.

Despite previous findings, it remains unclear through which mechanism the PPI-induced microbial changes contribute to an increased risk of infection and bacterial overgrowth in the intestines, as well as whether these PPI-associated effects are enhanced in old age. We hypothesized that the long-term administration of PPIs may induce alterations in the gut microbiota and butyrate levels, particularly in old age, which may be responsible for some adverse effects in host intestines. To test this hypothesis, we assessed the microbial alterations and butyrate levels following the long-term administration of PPIs to old F344 rats and investigated their associations with each other.

## Results

### Baseline characteristics

The total reads per sample was 122,643 ± 10,950 (mean ± SEM) (Supplementary Table 1). The number of operational taxonomic units (OTUs) per sample was 523.52 ± 57.92 (mean ± SEM). Good’s coverage of library (%) was 99.94 ± 0.01 (mean ± SEM).

No significant difference in body weight was observed among groups (Supplementary Fig. 1a). The rate of increase in body weight was significantly lower in the PPI group than in the control group for the 74-week-old rats (Supplementary Fig. 1b), while the rate of weight increase was not significantly different when comparing 2-year-old rats administered PPIs to the control group (Supplementary Fig. 1c).

### Clustering and phylum compositions

**Figure 1:**
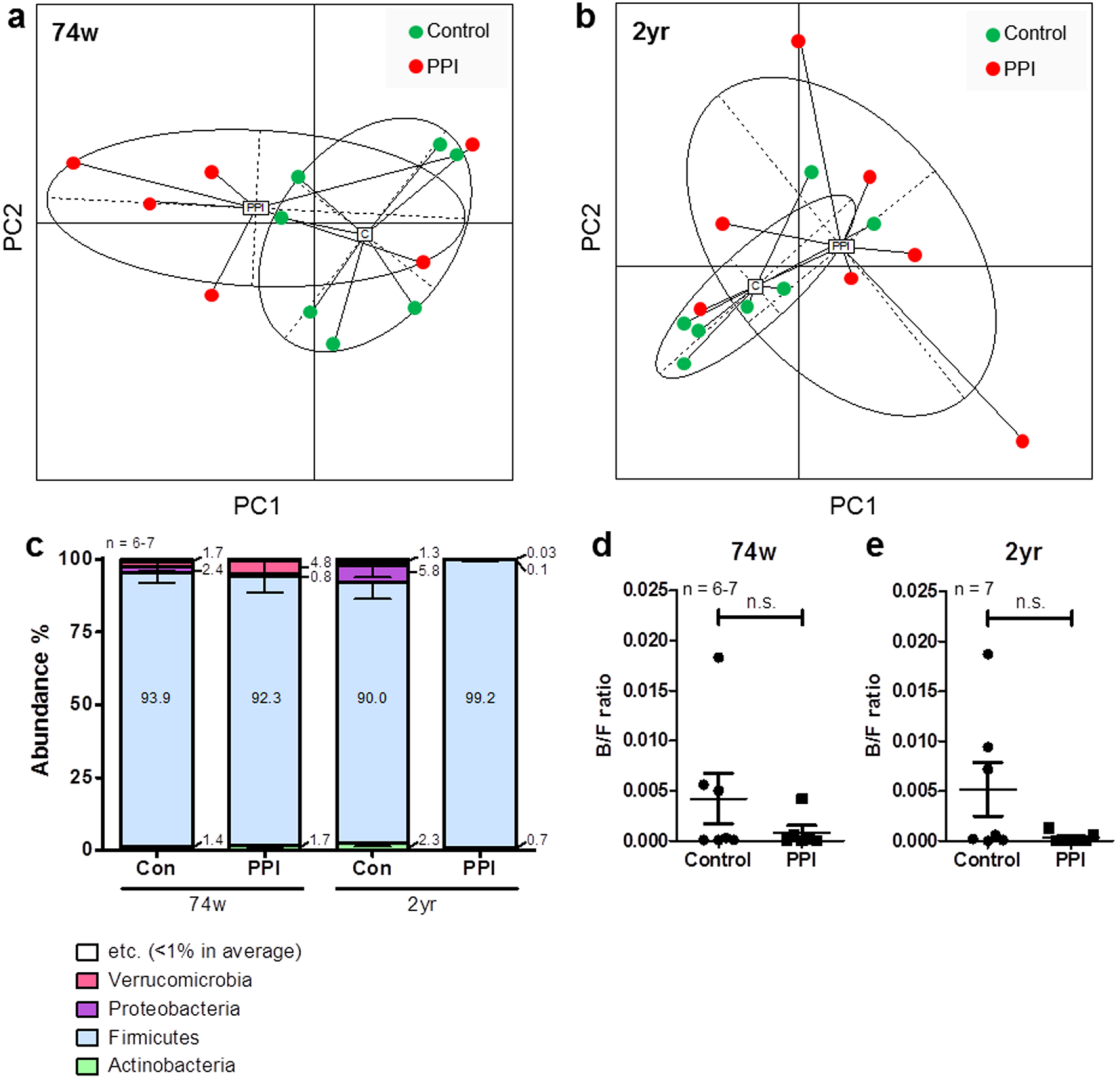
Sample clustering and phylum compositions. Clustering of samples of (**a**) 74-week-old and (**b**) 2-year-old rats by principal coordinates analysis (PCoA). (**c**) Taxonomic composition at the phylum level. Microbial composition of ileal contents from the control or PPI-administered 74-week-old and 2-year-old rats. Bacteroidetes/Firmicutes ratio of (**d**) 74-week-old and (**e**) 2-year-old rats. Data are expressed as the median, Q1 (25%), and Q3 (75%). Whiskers show the minimum and maximum values. PPI, proton pump inhibitor; n.s., not significant.

Figure 1 shows the clustering of samples by principal coordinates analysis (PCoA). No clear divergence was observed with respect to the administration of PPIs for both the 74-week-old and 2-year-old rats (Fig. 1).

Similarly, no significant effect of PPI on the abundance ratios at the phylum level was observed (Fig. 1c). In addition, the ratio of Bacteroidetes to Firmicutes was not significantly different between the control and PPI groups for both the 74-week-old and 2-year-old rats (Fig. 1d,e).

All OTU counts and abundance ratios are shown in Supplementary Dataset 1. All *P*-values and the false discovery rate (FDR) q-values obtained by comparing the control and PPI groups at each age are shown in Supplementary Dataset 2. The microbial compositions at the genus level are shown in Supplementary Fig. 2a–d. *Lactobacillus* was the most dominant genus in the 74-week-old control group, accounting for 40% on average (Supplementary Fig. 2a). In the 74-week-old PPI-administered rats, *Romboutsia* was the most prominent genus, accounting for 56% on average (Supplementary Fig. 2b). In contrast, *Turicibacter* was the most enriched genus in the 2-year-old control group, accounting for 29% on average (Supplementary Fig. 2c), while *Turicibacter* (47% on average) remained the most prominent genus in the 2-year-old PPI-administered rats but was increased compared to the control rats (Supplementary Fig. 2d). However, no significant differences in the abundances of the major genera (*Lactobacillus*, *Romboutsia*, and *Turicibacter*) were observed between control and PPI groups at each age (Supplementary Fig. 2e–j).

### Influence of PPIs on gut microbiota diversity

**Figure 2:**
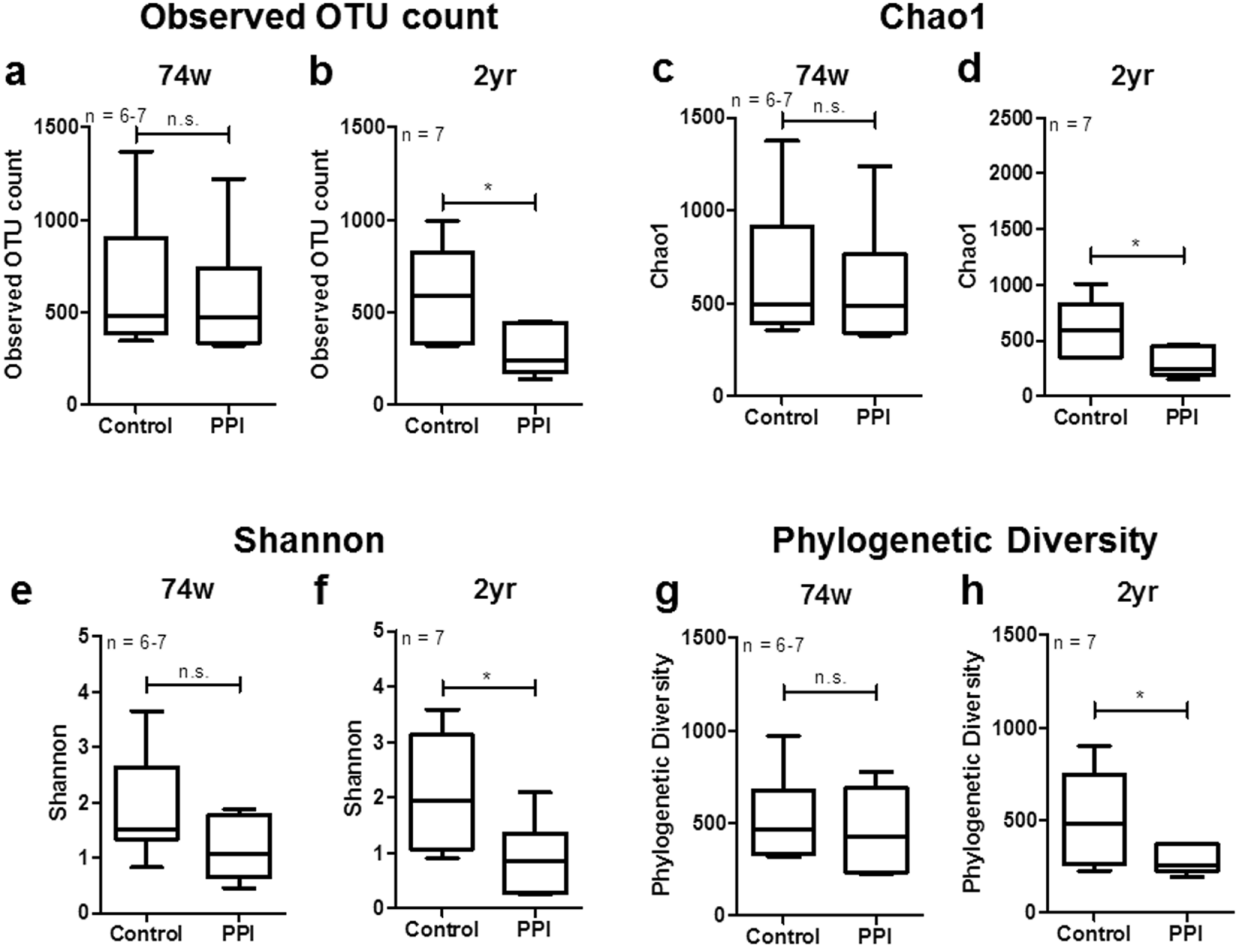
Alpha diversity of gut microbiota. (**a**,**b**) Observed OTU count, (**c**,**d**) species richness (Chao1), (**e**,**f**) alpha diversity (Shannon), and (**g**,**h**) phylogenetic diversity of the gut microbiomes of (**a**,**c**,**e**,**g**) 74-week-old and (**b**,**d**,**f**,**h**) 2-year-old rats. Data are expressed as the median, Q1 (25%), and Q3 (75%). Whiskers show the minimum and maximum values. The *P*-values for the Wilcoxon rank-sum test are shown in the figure; *P < 0.05 according to a Wilcoxon rank-sum test. PPI, proton pump inhibitor; n.s., not significant.

The observed OTU count significantly decreased in response to the PPI treatment in 2-year-old rats (*P* = 0.018) but not in 74-week-old rats (*P* = 0.668) (Fig. 2a,b). The species richness of the ileal microbiota, as indicated with Chao1 indices, was not significantly altered by the PPI treatment in the 74-week-old rats (Chao1: *P* = 0.668) (Fig. 2c). In contrast, in 2-year-old rats. the species richness (Chao1) was significantly decreased by the PPI treatment (Chao1: *P* = 0.018) (Fig. 2d). Similarly, no significant PPI-dependent alterations were observed in the alpha diversity (Shannon index) of the microbial community in the 74-week-old rats (*P* = 0.116), while the alpha diversity (Shannon) significantly decreased with the PPI treatment in 2-year-old rats compared to the control rats (*P* = 0.035) (Fig. 2e,f). Compared to the control rats, phylogenetic diversity significantly decreased in the 2-year-old rats (*P* = 0.048) but not the 74-week-old rats (*P* = 0.391) (Fig. 2g,h).

### Influence of PPIs on gut microbiota composition

**Figure 3:**
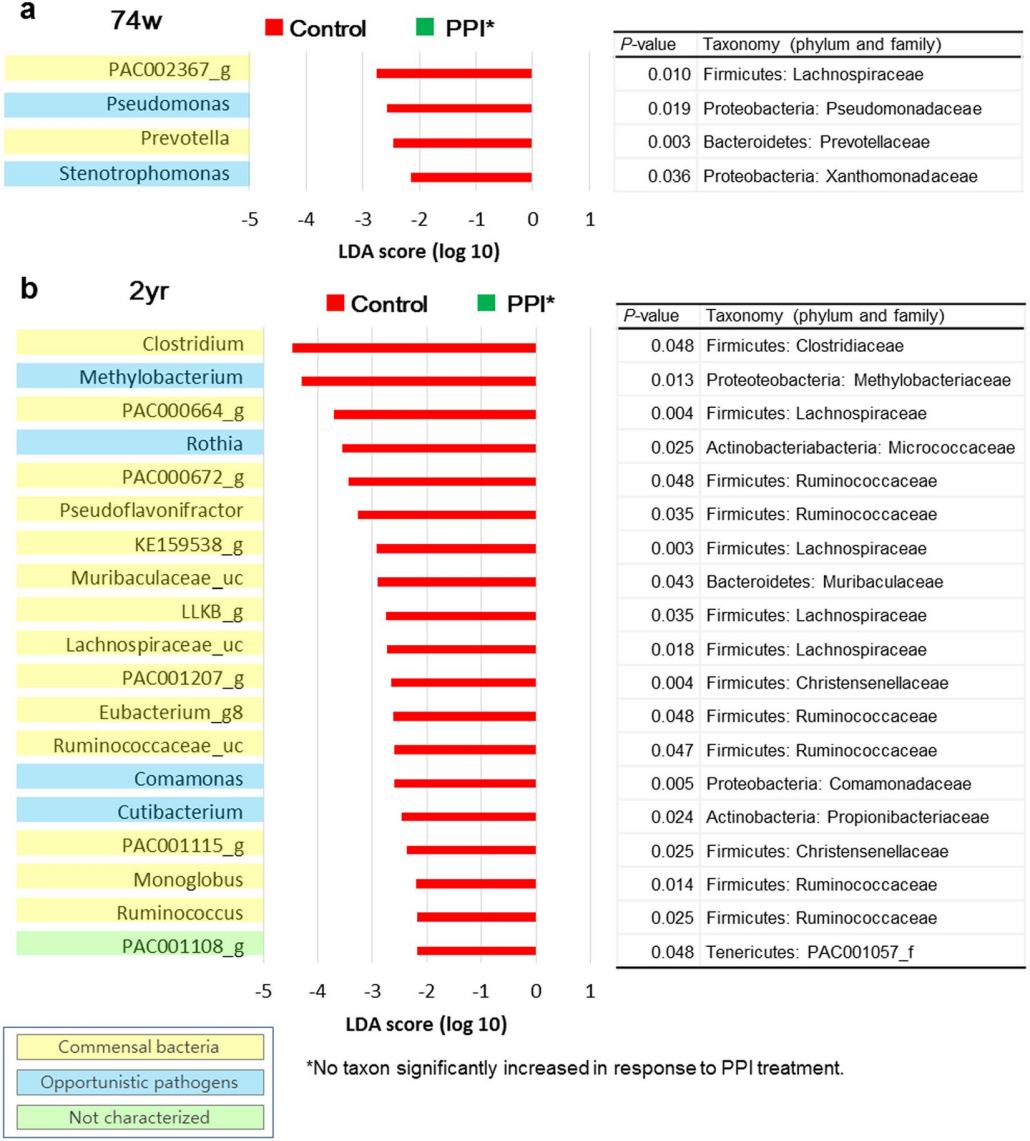
PPI-dependent alterations in the microbiota composition: linear discriminant analysis (LDA) effect size (LEfSe) analysis. Bar plots of genera with significant differences in abundance with LEfSe analysis in (**a**) 74-week-old (**b**) 2-year-old rats. Red bars show the LDA scores of genera that decreased by PPI treatment. No taxon significantly increased in response to PPI treatment, which was to be indicated with a green bar. Each colour on the genus name indicates the characteristics of each genus: yellow for commensal bacteria, blue for opportunistic pathogens, and green for not characterized bacteria. *P*-values were determined with the non-parametric factorial Kruskal-Wallis (KW) sum-rank test. The ‘Taxonomy (phylum and family)’ columns show the taxonomy of each taxon at the level of phylum and family. PPI, proton pump inhibitor; 74w, 74-week-old; 2 yr, 2-year-old.

According to the results of the linear discriminant analysis (LDA) effect size (LEfSe) analysis, some genera with minor proportions significantly increased or decreased in response to the PPI administration (Fig. 3). In the 74-week-old rats, the abundances of PAC002367_g (a genus of Lachnospiraceae) (*P* = 0.010), *Pseudomonas* (a genus of Proteobacteria) (*P* = 0.019), *Prevotella* (a genus of Bacteroidetes) (*P* = 0.003), and *Stenotrophomonas* (a genus of Proteobacteria) (*P* = 0.036) decreased with in response to the PPI treatment (Fig. 3a).

The abundance of the genus *Clostridium*, which belongs to the family Clostridiaceae in the phylum Firmicutes, was reduced as a result of the PPI treatment in 2-year-old rats (*P* = 0.048) (Fig. 3b). The genera *Methylobacterium* and *Comamonas*, belonging to the phylum Proteobacteria, were also diminished as a result of the PPI treatment in 2-year-old rats (*Methylobacterium*, *P* = 0.013; *Comamonas*, *P* = 0.005). Among genera of the families Lachnospiraceae and Ruminococcaceae, the abundances of PAC000664_g, PAC000672_g, *Pseudoflavonifractor*, KE159538_g, LLKB_g, Lachnospiraceae_uc, *Eubacterium*_g8, Ruminococcaceae_uc, *Monoglobus*, *Ruminococcus*, and PAC001108_g decreased with the PPI treatment in 2-year-old rats (all *P* < 0.05). The abundances of the genera *Rothia* and *Cutibacterium* of the phylum Actinobacteria, and the genus Muribaculaceae_uc of the phylum Bacteroidetes were reduced with the PPI treatment in 2-year-old rats (*Rothia*, *P* = 0.025; *Cutibacterium*, *P* = 0.024; Muribaculaceae_uc, *P* = 0.043). The genera PAC001207_g and PAC001115_g of the family Christensenellaceae, a part of the phylum Firmicutes, decreased with the PPI in 2-year-old rats (PAC001207_g, *P* = 0.004; PAC001115_g, *P* = 0.025).

As we analysed which species in *Clostridium* were influenced by PPI, we observed that the *Clostridium celatum* group and PAC001136_s were significantly affected by PPI (Supplementary Table 2). The *C. celatum* group includes *C. celatum*, *Clostridium disporicum*, *Clostridium saudiense*, and LAQZ_s, according to EzBioCloud, a taxonomically united database of 16S rRNA genes and whole genome assemblies^15^. *C. difficile* was not observed in this study (Supplementary Dataset 1).

### Enterotypes of microbiomes

**Figure 4:**
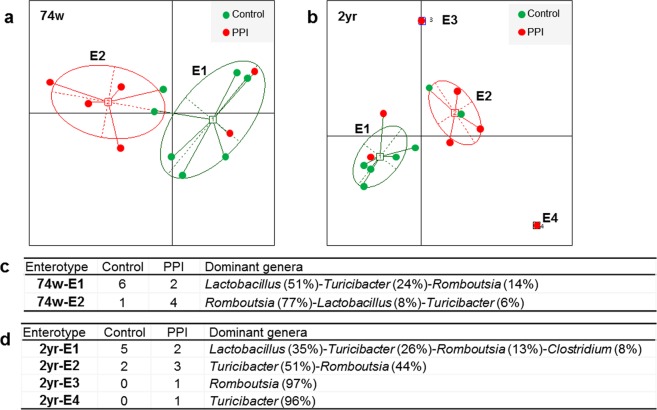
Enterotype clustering. (**a**) Two enterotypes (74w-E1, 2) of 74-week-old rat samples and (**b**) four enterotypes (2yr-E1, 2, 3, 4) of 2-year-old rat samples. The optimal cluster number was determined by maximizing the Calinski-Harabasz (CH) index value (Supplementary Fig. 3). The tables show the predominant genera in each enterotype of (**c**) 74-week-old and (**d**) 2-year-old rat samples. PPI, proton pump inhibitor.

To identify properties that were not shown in the comparison between groups in the LEfSe analysis, we separated the samples into enterotypes for each age group. The optimal cluster number was determined by maximizing the Calinski-Harabasz (CH) index value (Supplementary Fig. 3). Since the CH index was maximized when the cluster number was 2, samples of the 74-week-old rats were clustered into two enterotypes (Fig. 4a). In the 2-year-old rat samples, because the CH index was maximized when the cluster number was 4, samples from the 2-year-old rats were separated into four enterotypes (Fig. 4b). Enterotype 1 of 74-week-old rats was composed of six control samples and two PPI samples and was dominated by *Lactobacillus* (51% on average), followed by *Turicibacter* (24%) and *Romboutsia* (14%) (Fig. 4c). In contrast, enterotype 2 of 74-week-old rats comprised one control sample and four PPI samples and was dominated by *Romboutsia* (77%), followed by *Lactobacillus* (8%) and *Turicibacter* (6%) (Fig. 4c). Enterotype 1 of 2-year-old rats comprised five control samples and two PPI samples (Fig. 4d). The enterotype 1 of 2-year-old rats was dominated by *Lactobacillus* (35%), *Turicibacter* (26%), and *Romboutsia* (13%) (Fig. 4d). Enterotype 2 of 2-year-old rats comprised two control samples and three PPI group samples and was dominated by *Turicibacter* (51%) and *Romboutsia* (44%) (Fig. 4b,d). Enterotypes 3 and 4 of 2-year-old rats were identified in one PPI sample and were dominated by *Romboutsia* (97%) and *Turicibacter* (96%), respectively (Fig. 4d).

The alpha diversities of enterotypes 1 and 2 of 74-week-old rats were not significantly different (OTU count, q = 0.242; Chao1, q = 0.242; Shannon, q = 0.306; phylogenetic diversity, q = 0.306) (Supplementary Fig. 4a). Although the alpha diversity was significantly lower in enterotype 2 compared to enterotype 1 of 2-year-old rats, the phylogenetic diversity was not significantly different between these enterotypes (OTU count, q = 0.028; Chao1, q = 0.028; Shannon, q = 0.004; phylogenetic diversity, q = 0.570) (Supplementary Fig. 4b).

### Effect of PPIs on the concentration of butyrate in the intestinal tract

**Figure 5:**
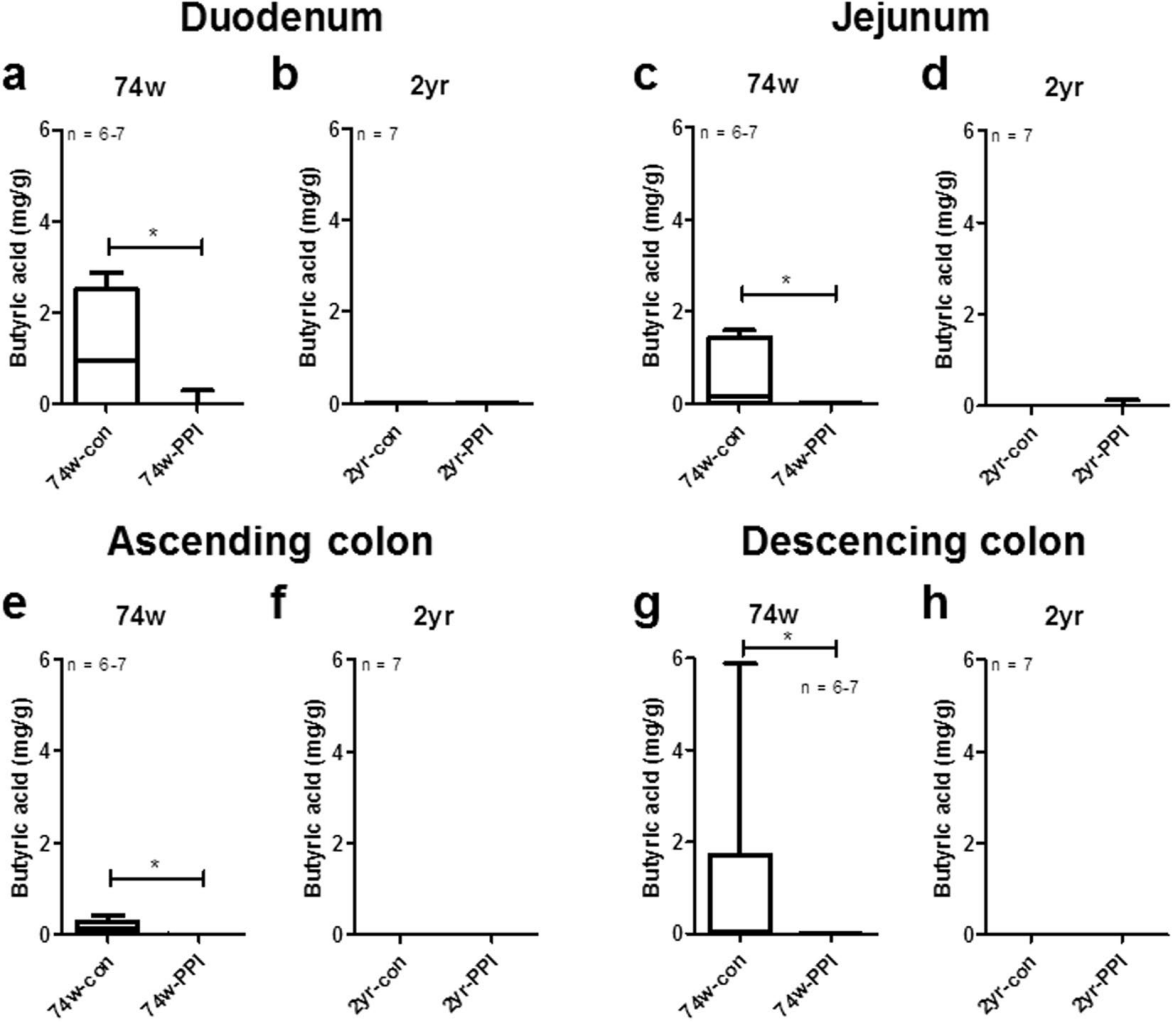
Concentration of butyrate in the duodenum, jejunum, ascending colon (AC) and descending colon (DC). Concentration (mg/g) of butyrate in the (**a**,**b**) duodenum, (**c**,**d**) jejunum, (**e**,**f**) AC and (**g**,**h**) DC of 74-week-old and 2-year-old rats. Data are expressed as median and range; *P < 0.05 according to a Wilcoxon rank-sum test. Con, control; PPI, proton pump inhibitor; 74w, 74-week-old; 2 yr, 2-year-old.

The levels of butyrate in the duodenum, jejunum, AC, and DC all significantly decreased with the PPI treatment in the 74-week-old rats but not in the 2-year-old rats (duodenum, 74w: *P* = 0.028, 2 yr: *P* = 1.000; jejunum, 74w: *P* = 0.009, 2 yr: *P* = 0.317; AC, 74w, *P* = 0.003, 2 yr, *P* = 1.000; DC, 74w, *P* = 0.025, 2 yr, *P* = 1.000) (Fig. 5).

### Correlations between butyrate concentration and *Lactobacillus* abundance

**Figure 6:**
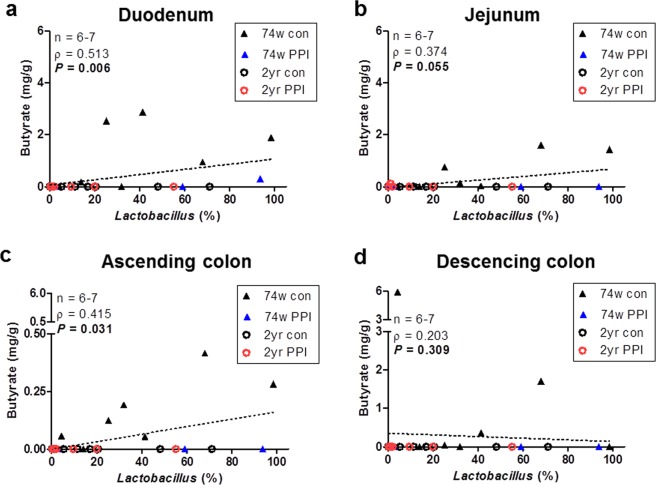
Correlations between abundance ratio (%) of *Lactobacillus* in ileum luminal contents and concentrations of butyrate in four intestinal regions. Correlations between the abundance of *Lactobacillus* (%) and butyrate concentrations in the (**a**) duodenum, (**b**) jejunum, (**c**) ascending colon, and (**d**) descending colon. Dotted line indicates the regression line. Spearman’s rho values and *P*-values were determined with the Spearman’s rank correlation coefficient method.

Since the enterotype analysis showed that most control samples belonged to enterotypes with high proportions of *Lactobacillus*, we analysed the correlation between the abundance of *Lactobacillus* in the ileum and the concentration of butyrate in each intestinal region. The results showed that the abundance of *Lactobacillus* was significantly and positively correlated with the concentration of butyrate in both the duodenum and the ascending colon (Fig. 6a,c). The abundance of *Lactobacillus* was also positively correlated with the butyrate level in the jejunum, but no significant difference was observed (*P* = 0.055). In the descending colon no significant correlation was observed (*P* = 0.309).

## Discussion

The results of multiple studies have demonstrated that PPIs affect the diversity or the composition of the gut microbiota^16–18^. In this study, we investigated the effects of the long-term use of PPIs on the ileal microbiome and microbial fermentation products in old rats. The results of this study showed a clear effect of PPIs on the gut microbiota and butyrate concentrations in old rats.

First, we observed that the long-term administration of PPIs induced alterations in microbial diversity and compositions exclusively in rats with an older age. The administration of PPIs to rats of an older age induced more severe alterations in microbiota diversity, which may increase the risk of infection. This result strongly suggests that ageing affects the resistance of microbiota to external stimuli, which is consistent with a previous report showing that the resistance of the gut microbiota to high-fat diet was weaker in older rats^8^. Under normal conditions, the intestinal microbiota inhibits growth of pathogenic bacteria through colonization resistance^19^. However, a decreased diversity reduces the ability of the gut microbiota to suppress bacterial infections. For instance, a reduced diversity of gut microbiota is a feature of dysbiosis in *C. difficile* infections^20^. In this study, no *C. difficile* was observed in the microbiota; however, the reduced diversity may increase the risk of *C. difficile* infection. This explanation is consistent with a previous study which showed that receipt of antibiotics suppresses the normal gut microbiota, and thereby provides a “niche” for *C. difficile* infection^21^. Consistently, an increase of bacterial diversity through faecal microbiota transplantation has been suggested as a potential therapeutic method for some disorders, including pseudomembranous colitis and inflammatory bowel diseases^22^. Therefore, PPI use during old age may induce more remarkable changes in the gut microbiota than in younger individuals, and these changes would result in an increased risk of bacterial infection and microbial dysbiosis-related diseases.

Along with the decreased diversity, PPIs induced alterations in microbial compositions. The growth of commensal bacteria decreased in the guts of old rats administered PPIs. Within the phylum Bacteroidetes, two genera were altered by the PPI treatment, *Prevotella* and Muribaculaceae_uc. Members of the genus *Prevotella*, which decreased with the PPI treatment in 74-week-old rats, are associated with carbohydrate consumption and may be involved in osteomyelitis. The abundance of Muribaculaceae_uc, which was reduced with the PPI treatment in 2-year-old rats, is a dominant bacterium in the mouse gut^23^. In addition, the abundances of two genera of the family Christensenellaceae, PAC001207_g and PAC001115_g, were observed to be altered with the PPI treatment in the present study*. Christensenella minuta*, *Christensenella timonensis*, and *Christensenella massiliensis* are the most studied genera of the family Christensenellaceae, all of which are isolated from human faeces. Christensenellaceae is associated with a low body mass index (BMI), as observed in a twins study and is related to a low body weight gain in mice^24^. The suppression of growth of these commensal bacteria by PPI treatment may lead to overgrowth of certain genera. In this study, the majority of the rats treated with PPI were dominated by *Turicibacter* or *Romboutsia*, as shown in Fig. 4d.

It appears that the PPIs reduce the growth of some opportunistic pathogens as well as commensal bacteria. The abundance of the genera *Pseudomonas* and *Stenotrophomonas*^25^, the opportunistic human pathogens, decreased with the treatment of 74-week-old rats with PPIs. Similar to the reduction of *Pseudomonas* and *Stenotrophomonas*, in 2-year-old PPI-administered rats, the abundances of some opportunistic pathogens, including *Methylobacterium*, *Comamonas*, *Rothia* and *Cutibacterium*, were reduced in 2-year-old rats. In humans, the abundance of Methylobacteriaceae has been observed to decrease in the stomach in response to PPI treatment^6^, which is consistent with our results. However, the observed decrease of Comamonadaceae in our study was inconsistent with human studies showing an increase of Comamonadaceae in the oesophagus^6^. We presume that regional differences caused the disparity between ours and previous results. Among *Clostridium* spp., *C. celatum* most prominently decreased with the PPI treatment. Regarding the possibility of *C. celatum* pathogenicity in humans^26^, the reduction of *C. celatum* with the PPI treatment may be a beneficial aspect of PPI-induced microbial changes. Although the PPI administration has been shown to reduce the growth of some opportunistic pathogens, the lack of a diverse bacterial community may promote the overgrowth of infectious bacteria^20^, as described in the discussions regarding diversity changes.

We examined the effect of PPI administration on the concentration of butyrate in various intestinal regions of old rats and estimated correlations with microbial components affected by PPI. Our results show that the PPI treatment decreased the butyrate levels in four different regions of small and large intestines and that butyrate levels were depleted in the 2-year-old control rats (Fig. 5). In a previous report, no significant change in butyrate was observed after administration of omeprazole in mice for two weeks^27^. However, this previous study did not examine the effects of PPIs in old mice, and the period of PPI administration was very short in comparison to the present study. In this study, we suggest that the long-term administration of PPIs during old age may reduce the butyrate levels in the duodenum, jejunum, ascending and descending colons. Butyrate contributes to host health through modulation of gene expression and the regulation of the growth of some bacteria^28^, in addition to its anti-inflammatory and anti-proliferative properties in colons^29^. Therefore, the decreased level of butyrate in PPI-administered small and large intestines may contribute to the induction of the adverse effects of PPIs, such as pseudomembranous colitis.

According to the enterotype analysis, we demonstrated that the abundance of *Lactobacillus* decreased following PPI administration, which significantly correlated with the concentration of butyrate. *Lactobacillus* spp. have been widely used as probiotics. *Lactobacillus acidophilus* has been shown to increase the level of butyrate in the large intestines of pigs^30^, and this bacterium stimulates butyrate uptake by colonocytes through the regulation of monocarboxylate transporter 1 (MCT1) expression^31^. Therefore, changes in the microbiome into low-*Lactobacillus* enterotypes following the long-term use of PPIs may be involved in the decreased concentrations of butyrate in PPI-administered old rats. In addition, most of the 2-year-old PPI-administered rats (5 out of 7) experienced enrichment of *Turicibacter* and/or *Romboutsia*, which indicates the promotion of dysbiosis by PPIs in old age. This result is consistent with that of a previous study that suggested that *Turicibacter* may play a role in inflammatory bowel diseases^32^. The dominance of *Romboutsia* by PPI treatment was observed in both 74-week-old and 2-year-old rats; however, the pathogenicity of *Romboutsia* has yet to be confirmed.

In this study, there was no clear separation of samples by PPI according to principal coordinate analysis (PCoA). However, we observed individual variations in the influence of PPIs in humans^33^. We presume that this variation is consistent in animals. LEfSe analysis was suitable for identifying taxa associated with a specific factor (PPI in this study)^34^; however, the enterotype analysis additionally identified genera altered by PPI, *Lactobacillus* and *Romboutsia*, by excluding the effects of outliers. Therefore, we were able to determine the effects of PPIs more thoroughly by using both of these analytical methods.

Besides the gut microbial changes, the PPI-mediated changes in the gut environment can increase the infection risk by affecting the gastric acidity and the immune systems^35^. Firstly, elevated gastric pH results in the increased bacterial colonization in the stomach^36^. This effect might be more severe in the aged since the gastric acid secretion decreased with age in rats^37^. In addition, chronic PPI treatment may reduce the function of neutrophils that produce reactive oxygen species, through the increase of the basal cytosolic calcium concentrations in the neutrophils^38^. This influence of PPI consequently impairs the bactericidal activity of neutrophils. These first-line alterations in the gut environment influence on both the enteric infection risk and the gut microbial alterations. The present study has significance that it has characterized the consequent changes in the gut microbiota and their metabolites.

This study however had limitations. First, the microbial changes before and after the PPI administration were not compared. Despite this limitation, the results of the present study are important in that the microbiota among the old animals were compared, depending on the long-term administration of PPI. Second, we were unable to measure the butyrate levels in the ileum, primarily because we used all of the ileum contents for the NGS analysis. Third, the butyrate levels in 2-year-old rats were under the detection limit of the method we employed in this study (0.05 mg/g). Third, the butyrate levels in 2-year-old rats were under the detection limit of the method we employed in this study (0.05 mg/g). However, as the data of 74-week-old rats became very low we interpreted that the levels of 2-year-old rats were further low to be detected. Fourth, the female rats were not included. However, according to previous results, it seems that the diversity of the microbiome does not significantly differ depending on sex^8,39^. There have been some reports of sex differences in the gut microbial compositions. However, since the increase of gastric pH by PPI, the major alteration by PPI, is consistent in both males and females, we presumed that there would be not so much differences depending on sex, regarding the effect of PPI on gut microbiota. Fifth, the influences of PPI on the immune responses of the rats were not analyzed. PPI-mediated changes in the immune responses and gastric acidity may be associated with the microbial alterations shown in this study.

In conclusion, the long-term use of PPIs alters the gut microbiota and butyrate concentrations, particularly in old age, which could be an underlying mechanism of PPI-induced adverse effects, such as pseudomembranous colitis.

## Methods

### Animals and sample collection

Specific-pathogen-free F344 male rats (74-week-old and 2-year-old) were used as an ageing animal model (Orient Bio, Seongnam, Korea)^40,41^. Each animal was housed in a cage maintained at 23 °C with an alternating 12-hour light/dark cycle under specific pathogen-free conditions. Experiments were conducted in four groups according to age and diet (n = 6–7 in each group): 74-week-old chow-fed (n = 7), 74-week-old PPI-fed (n = 6), 2-year-old chow-fed (n = 7), and 2-year-old PPI-fed groups (n = 7). One sample of the 74-week-old PPI-fed group was excluded for being an outlier according to the PCoA. Beginning at 24 or 54 weeks of age, rats in the chow diet groups were fed a normal chow diet *ad libitum* for 50 weeks. Usually, PPI is treated in adulthood in human for peptic ulcer disease and gastroesophageal reflux disease. Thus we decided the PPI supplementation for 50 weeks, to observe the effects of long-term administration of PPI beginning from the young adulthood or middle-age, sustaining until the old age. The corresponding age in rats was 24-week (young adulthood in humans) or 54-week-old (middle-age in human), which reach on 74-week-old (approximately 60 years in humans) or 2-year-old (approximately 80 years in humans) at the end of PPI administration. Actually we have performed a preliminary study treating PPI from 24-week-old to 74-week-old^10^. In the present study we decided to extend this age until 2 year old because PPI is frequently used in the old age using 24-week or 54-week-old rats (administered with PPI until they reach on 74-week or 2-year-old, respectively).

Rats in the PPI-supplemented diet groups were fed the same chow diet supplemented with lansoprazole (5 mg/kg (body weight)/day). One gram of lansoprazole was mixed with 20 kg of chow to obtain the daily dose. Terminal anaesthesia was conducted via inhalation of carbon dioxide. A 1-cm length of the terminal ileum was obtained from each rat, and phosphate-buffered saline was flushed through the lumens. The flushed material was centrifuged at 20,000 × g for 30 minutes to pellet the bacteria. The pellet was processed to extract the total microbial DNA using a commercial kit (iNtRON Biotechnology, Seongnam, Korea). This study was performed in accordance with the recommendations of the Guide for the Care and Use of Laboratory Animals of South Korea. The protocol was approved by the Institutional Animal Care and Use Committee (IACUC) of the Seoul National University Bundang Hospital (Permission No. BA1304-127/033-07).

### Metagenome sequencing

The V3-V4 region of the 16S rRNA gene was amplified by PCR using the primers 341 F (5′-TCGTCGGCAGCGTCAGATGTGTATAAGAGACAGCCTACGGGNGGCWGCAG-3′) and 805 R (5′-GTCTCGTGGGCTCGGAGATGTGTATAAGAGACAGGACTACHVGGGTATCTAATCC-3′). The PCR products were confirmed by electrophoresis and were purified using a QIAquick PCR purification kit (Qiagen, Valencia, CA, USA). The purified PCR products were tagged with Illumina indices and adapters from a Nextera® XT Index Kit (Illumina, San Diego, CA, USA). Short DNA fragments were eliminated using a FavorPrep™ DNA purification kit (Favorgen, Taiwan). The PCR amplicons were quantified using a Quant-iT™ PicoGreen™ dsDNA Assay Kit (Thermo Fisher Scientific, Wilmington, DE, USA). After pooling 300 ng of DNA per sample, the PCR products were purified with a FavorPrep™ DNA gel extraction kit (Favorgen, Taiwan). The quality assessment for confirmation of DNA integrity and product size was conducted on a Bioanalyzer 2100 instrument (Agilent, Palo Alto, CA, USA) using a DNA 7500 chip at ChunLab, Inc. (Seoul, South Korea). Metagenome sequencing was performed using the Illumina MiSeq platform at ChunLab, Inc. (Seoul, South Korea).

The raw reads were processed starting with the quality check and filtering of low quality (<Q25) reads using Trimmomatic 0.32^42^. The paired-end sequence data was merged using PANDAseq^43^. The primer sequences were then trimmed with an in-house programme of the ChunLab, Inc. (Seoul, South Korea) at a similarity cut off of 0.8. Non-specific amplicons that did not encode 16S rRNA were identified by using the HMMER program hmmsearch with 16S rRNA profiles^44^. Sequences were denoised using DUDE-Seq^45^, and non-redundant reads were extracted through UCLUST-clustering^46^. The EzBioCloud database was utilized for taxonomic assignment using USEARCH (8.1.1861_i86linux32)^46^ followed by more precise pairwise alignment^47^. UCHIME^48^ and the non-chimeric 16S rRNA database from EzBioCloud were used to detect chimaeras for reads that contained less than a 97% best hit similarity rate. The sequence data was then clustered using CD-HIT^49^ and UCLUST^46^.

To visualize the sample differences, PCoA was performed with unweighted UniFrac^50^. PCoA plots were generated using the ade4 package and s.class function in R. The clustering of samples was explained with the principal coordinate (PC) values. In addition, an Unweighted Pair Group Method with Arithmetic mean (UPGMA) tree was created using BIOiPLUG (ChunLab, Inc.). Beta diversity distances were calculated using Generalized UniFrac. Microbial diversity (Observed OTU count, Chao1, Shannon indices, and phylogenetic diversity) was examined using BIOiPLUG. Taxonomic summary bar charts were created for the OTU abundance ratio (%) at the phylum and family levels using GraphPad Prism (version 5.01). Enterotype stratification was identified in the ileum content samples using the ClusterSim function in R. The optimal number of clusters was determined by maximizing the value of the CH index^51^.

### Linear discriminant analysis (LDA) effect size (LEfSe) analysis

A LEfSe analysis (http://huttenhower.sph.harvard.edu/galaxy/) was conducted to determine the taxonomic compositions that were significantly changed by the PPI treatment^34^. The conditions for the LEfSe analysis were: (1) an alpha value for the factorial Kruskal-Wallis test between the control and the PPI treatment groups of less than 0.05; (2) an alpha value for the pairwise Wilcoxon test among the taxonomic compositions of less than 0.05; (3) a threshold of the logarithmic LDA score for discriminative features of less than 2.0; and (4) a multi-class analysis set as all-against-all.

### Measurements of butyrate concentrations

Contents of duodenum, jejunum, ascending colon, and descending colon were extracted as described previously^52^, and the concentration of butyrate in each extract was measured via high-performance liquid chromatography (HPLC). Briefly, distilled water was added to 20–50 mg of each intestinal content to a final weight of 200 mg. Following incubation at 80 °C for 15 min, each sample was centrifuged at 13,000 rpm for 10 min, after which the supernatant was filtered through a membrane filter (pore size 0.45 μm). Butyrate in each sample was then separated and measured using an Agilent 1100 series instrument (Agilent, CA, USA) equipped with a C18 column (ZORBAX Eclipse XDB-C18, analytical 4.6 * 150 mm, 5-Micron, Agilent, CA, USA) and a UV detector (210 nm). The mobile phase consisted of 90% 10 mM KH_2_PO_4_ and 10% acetonitrile. The detection limit of the HPLC of butyrate was 0.05 mg/g.

### Statistical analysis

Statistical calculations other than for pyrosequencing data were performed using PASW Statistics version 18.0 (SPSS Inc., 2009, Chicago, IL, USA). The two groups were compared by the Wilcoxon rank-sum test, and p-values < 0.05 were considered to be significant. For the adjustment of multiple comparison, false discovery rate (FDR)-corrected q-values were calculated with a significance threshold of 5%, if needed.

## Supplementary information

Supplementary Information

Supplementary Dataset 1

Supplementary Dataset 2

## Data Availability

The raw datasets generated during the current study are available in the NCBI Sequence Read Archive (SRA accession number SRR8357186 ~ SRR8357213), https://trace.ncbi.nlm.nih.gov/Traces/sra/sra.cgi?view=run_browser&run. The processed data generated and analysed during this study are included in this published article as its Supplementary Dataset files.
