# Supplementary material for: Gut microbiota and butyrate level changes associated with the long-term administration of proton pump inhibitors to old rats
Source: Sci Rep. 2019 Apr 29;9:6626. doi: 10.1038/s41598-019-43112-x (PMC6488615; doi:10.1038/s41598-019-43112-x)
Supplement: Supplementary file 1 — Supplementary Information [file 41598_2019_43112_MOESM1_ESM.docx]

Supplementary Information

RE: Gut microbiota and butyrate level changes associated with the long-term administration of proton pump inhibitors to old rats

Sun Min Lee^1^, Nayoung Kim^1,2*^, Ryoung Hee Nam^1^, Ji Hyun Park^2^, Soo In Choi^1^, Young-Tae Park^3,4^, Yeon-Ran Kim^3^, Yeong-Jae Seok^3^, Cheol Min Shin^1^, Dong Ho Lee^1,2^


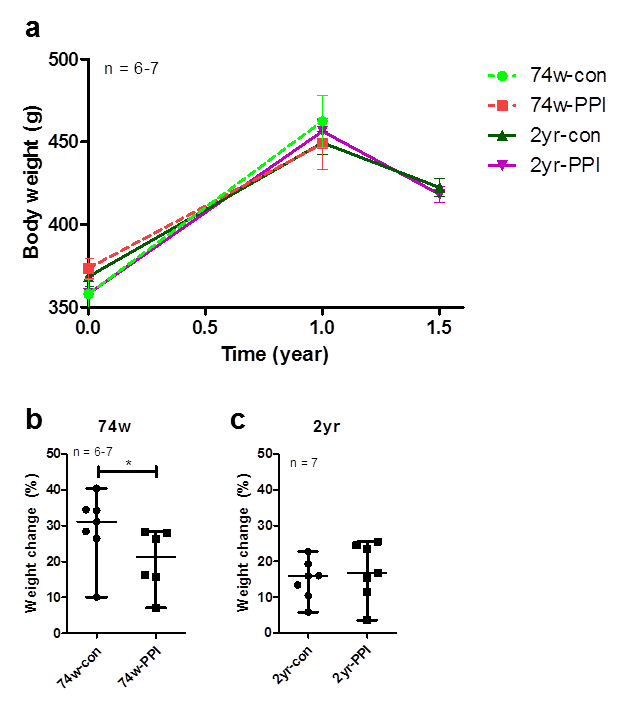


**Supplementary Figure 1. Change in body weight.** (a) Body weight during the experiment. Means and SEM. Weight change rates (%) of (b) 74-week-old and (c) 2-year-old rats. Median and range. Con, control; PPI, proton pump inhibitor.

**
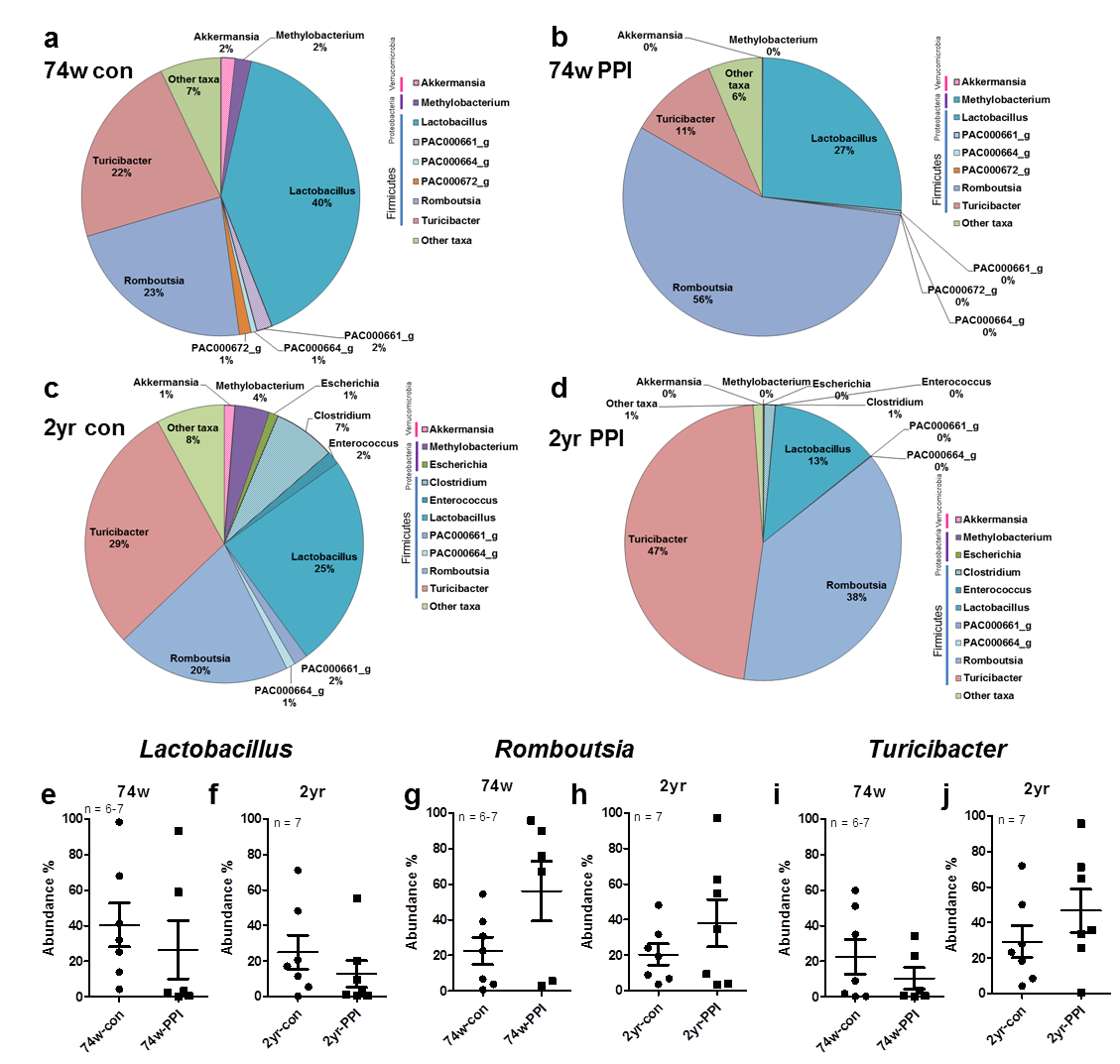
**

**Supplementary Figure 2. Taxonomic composition at the genus level.** Microbial composition of the ileal contents from 74-week-old (a) control and (b) PPI groups and from 2-year-old (c) control and (d) PPI groups. Abundance ratios of dominant genera, including (e,f) *Lactobacillus*, (g,h) *Romboutsia*, and (i,j) *Turicibacter.* Pie charts show the mean values, and scatter plots show the means and SEM. PPI, proton pump inhibitor.

**
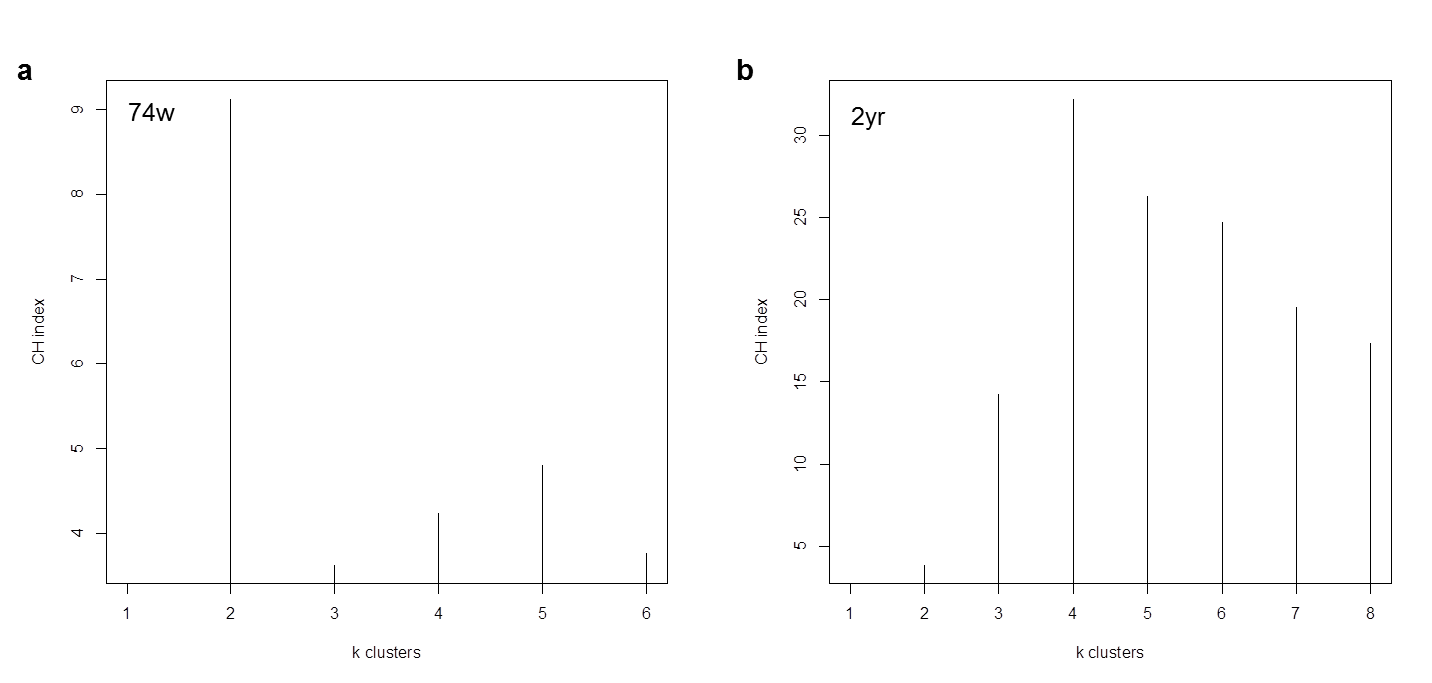
**

**Supplementary Figure 3. Calinski-Harabasz (CH) index for the separation of enterotypes.** Enterotypes were determined based on the CH index. (a) Samples from 74-week-old rats were separated into two enterotypes based to the highest CH index when k cluster was 2. (b) The CH index of the 2-year-old rat samples was highest when k clusters was 4.


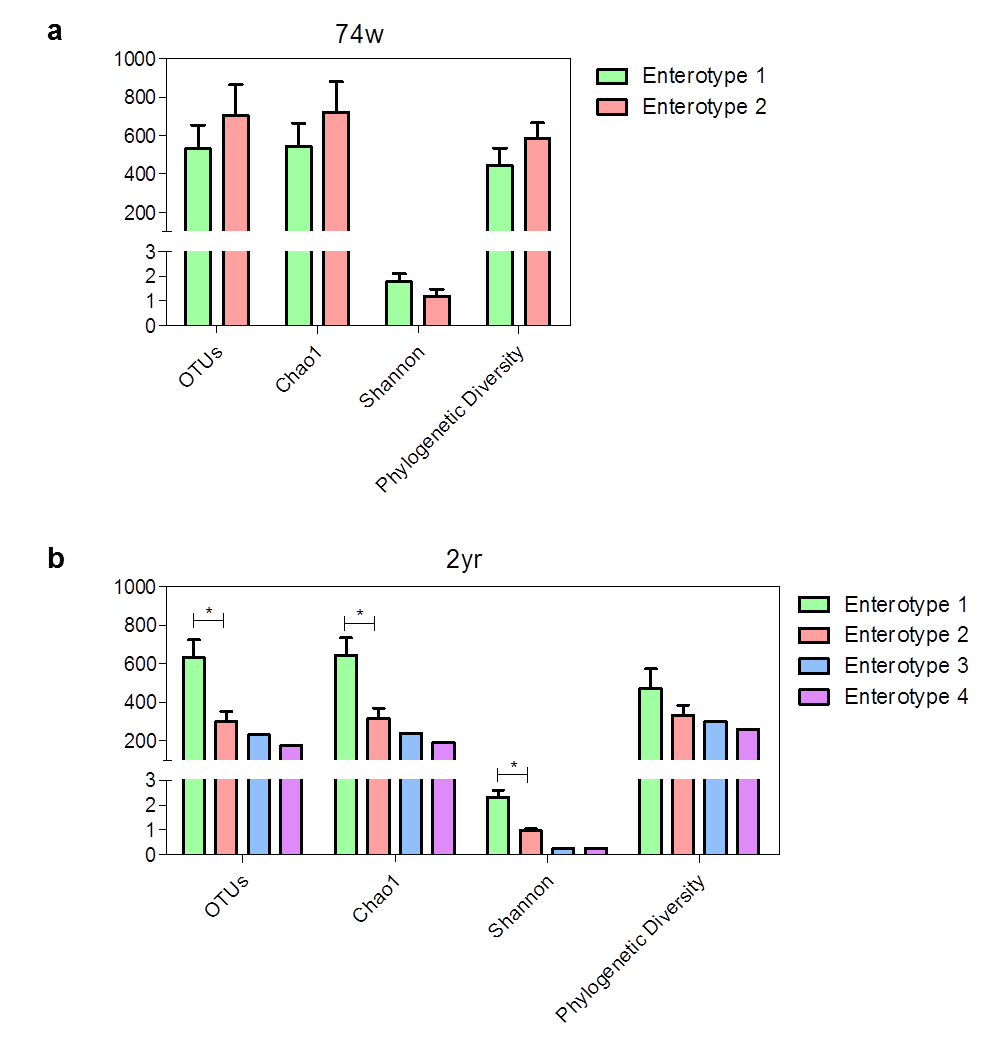


**Supplementary Figure 4. Alpha diversity in enterotypes.** Number of OTU counts and values of Chao1 index, Shannon index, and phylogenetic diversity values for (a) 74-week-old and (b) 2-year-old rats. Means and SEM. 74w, 74-week-old rats; 2yr, 2-year-old rats. ^*^q < 0.05 by false discovery rate of *P*-values by Wilcoxon rank-sum test.

**Supplementary Table 1.** Baseline characteristics of 16S rRNA metagenome sequencing data and alpha, beta diversity of microbiota from ileum luminal contents.

| Characteristics | 74w control  (n = 7) | 74w PPI  (n = 6) | *P*-value^a^ | 2yr control  (n = 7) | 2yr PPI  (n = 7) | *P*-value^a^ |
| --- | --- | --- | --- | --- | --- | --- |
| Valid reads | 101,001 | 117155 | 0.886 | 83,849 | 142,810 | 0.180 |
| No. of OTUs | 480 | 472 | 0.668 | 593 | 238 | **0.018** |
| Good’s library coverage (%) | 99.94 | 99.95 | 0.475 | 99.93 | 99.97 | **0.048** |
| Alpha diversity |  |  |  |  |  |  |
| ACE | 519.99 | 506.04 | 0.668 | 616.59 | 261.18 | **0.018** |
| Chao1 | 493.88 | 485.03 | 0.668 | 598.34 | 247.76 | **0.018** |
| Jackknife | 549 | 536 | 0.775 | 646 | 279 | **0.018** |
| Shannon | 1.510 | 1.072 | 0.116 | 1.954 | 0.870 | **0.035** |
| Simpson | 0.323 | 0.598 | 0.063 | 0.207 | 0.514 | 0.064 |
| Beta diversity^b^  (Inter-set distances to control) | 0.688 | 0.674 | 0.339 | 0.617 | 0.584 | 0.111 |

^a^Wilcoxon rank-sum test. ^b^Generalized UniFrac. Data are presented as the medians. PPI, proton pump inhibitor. Boldface indicates significance.

**Supplementary Table 2.** *Clostridium* species that were altered by long-term administration of PPIs in the ileal microbiota of 2-year-old rats.

| Taxon name | Taxonomy | LDA effect size | p-value | p-value (FDR) | Abundance ratio (mean) | |
| --- | --- | --- | --- | --- | --- | --- |
|  |  |  |  |  | 2yr-control | 2yr-PPI |
| *Clostridium celatum* group | Bacteria: Firmicutes: Clostridia: Clostridiales: Clostridiaceae: *Clostridium* | 4.462 | 0.048 | 0.051 | **7.059** | **1.262** |
| PAC001136_s | Bacteria: Firmicutes: Clostridia: Clostridiales: Clostridiaceae: *Clostridium* | 2.275 | 0.025 | 0.026 | 0.042 | 0.005 |

Boldface indicates a genus with an abundance ratio > 1% on average. LDA effect size, Linear discriminant analysis (LDA) effect size; FDR, false discovery rate; PPI, proton pump inhibitor.

**Supplementary Dataset 1.** (The data file is supplied as a separate file: Supplementary_Dataset_1.xlsx.) Operational taxonomic unit (OTU) counts and abundance ratios of each taxon. con, control; PPI, proton pump inhibitor.

**Supplementary Dataset 2.** (The data file is supplied as a separate file: Supplementary_Dataset_2.xlsx.) Mann-Whitney U values, Wilcoxon W values, Z values, *P*-values, and q-values (false discovery rate) of every taxon in gut microbiota of 74-week-old and 2-year-old rats. *P*-values were determined with the Wilcoxon rank-sum test. q-values were determined by false discovery rate of the *P*-values by Wilcoxon rank-sum test. con, control; PPI, proton pump inhibitor; FDR, false discovery rate.
